# Coronary Computed Tomography Angiography in Heart Transplant Patients: Current Insights and Future Directions

**DOI:** 10.1097/TP.0000000000005266

**Published:** 2025-05-18

**Authors:** Britt C.J. van Dijk, Daniel Bos, Stefan Roest, Alexander Hirsch, Yannick J.H.J. Taverne, Jasper J. Brugts, Rudolf A. de Boer, Ricardo P.J. Budde, Olivier C. Manintveld

**Affiliations:** 1 Department of Cardiology, Thorax Center, Cardiovascular Institute, Erasmus MC, University Medical Center Rotterdam, Rotterdam, the Netherlands.; 2 Erasmus MC Transplant Institute, Erasmus MC, University Medical Center Rotterdam, Rotterdam, the Netherlands.; 3 Department of Radiology and Nuclear Medicine, Erasmus MC, University Medical Center Rotterdam, Rotterdam, the Netherlands.; 4 Department of Epidemiology, Erasmus MC, University Medical Center Rotterdam, Rotterdam, the Netherlands.; 5 Department of Cardiothoracic Surgery, Thorax Center, Erasmus MC, University Medical Center Rotterdam, Rotterdam, the Netherlands.

## Abstract

Cardiac allograft vasculopathy (CAV) remains a significant challenge after heart transplantation, necessitating effective surveillance methods. This review centers around the role of coronary computed tomography angiography (CCTA) in CAV surveillance, given its unique capabilities to visualize and quantify CAV in comparison with other imaging modalities, including invasive coronary angiography and intravascular ultrasound. CCTA has shown good diagnostic performance for detecting and monitoring CAV, exemplified by a higher sensitivity and negative predictive value compared with invasive coronary angiography. Additionally, CCTA can provide valuable functional insights with fractional flow reserve integration. An additional, considerable benefit of CCTA is that it allows for the opportunity to assess other imaging markers of cardiometabolic and general health, including coronary artery calcium score, epicardial fat volume, liver fat, vertebral bone density, and lung density, which allows for a comprehensive assessment of the overall health of the patient.

## INTRODUCTION

Cardiac allograft vasculopathy (CAV) remains a leading cause of chronic graft failure and death after heart transplantation (HTx).^1,2^ Almost half of HTx recipients will develop CAV within 10 y after transplantation,^1-4^ and the chances of graft failure increase if a recipient develops CAV within 3 y after HTx.^2,4,5^ CAV has distinct features, which sets it apart from traditional coronary atherosclerosis in non-HTx patients. Coronary atherosclerosis is a dynamic disease that usually develops over decades and mostly affects the proximal coronary vessels. Atherosclerotic plaques are characterized as focal, often with deposition of calcium, thus giving eccentric proliferation of the intima.^1,3,6-8^ In contrast, CAV is a diffuse disease affecting the entire vasculature of the allograft, often beginning with the microvasculature. CAV can develop as early as 1 or 2 y after HTx but may also manifest later, beyond 2y. The rate of CAV progression and the timing after transplantation are key factors influencing adverse outcomes.^1,6,8,9^ Moreover, signs of inflammation are usually present with accompanying endothelial damage. CAV-related wall thickening is diffuse along the whole length of the coronaries and is concentric, which is typically known as concentric intimal hyperplasia. Finally, the CAV lesions are generally devoid of calcium.^1,6,8^ Notably, CAV lesions and atherosclerotic plaques can occur simultaneously in HTx patients.^10^

There is a need to identify and follow up on CAV in HTx. Repeated coronary angiography is a burden for patients, costly, as well as associated with complications. Nowadays, as in atherosclerosis, most attention is focused on the role of computed tomography (CT) scanning to visualize CAV. Therefore, in this review, we will evaluate the current recommendations for CAV surveillance, highlight the opportunities of CT-based surveillance, and benchmark this against the current recommendations for assessment of the coronary arteries and other cardiometabolic health markers. Additionally, we will briefly highlight other techniques that may be used for CAV surveillance, but given their costs or invasiveness are less likely to be incorporated as standard practice, but could prove to be resourceful given specific circumstances.

## CURRENT RECOMMENDATION: CORONARY ANGIOGRAPHY AND INTRAVASCULAR ULTRASOUND

In 2010, The International Society for Heart and Lung Transplantation (ISHLT) standardized the classification of CAV and introduced invasive coronary angiography (ICA), with or without intravascular ultrasound (IVUS) as the gold standard for CAV surveillance because of its good accessibility.^1,2,6,8^ The optimal frequency for surveillance of CAV is not well defined but is typically performed every 1 or 2 y.^11^ Since then, possibilities for noninvasive imaging-based surveillance, especially with the increasing use of CT, have expanded, and this has prompted a call for new recommendations.^11-13^

**FIGURE 1.**
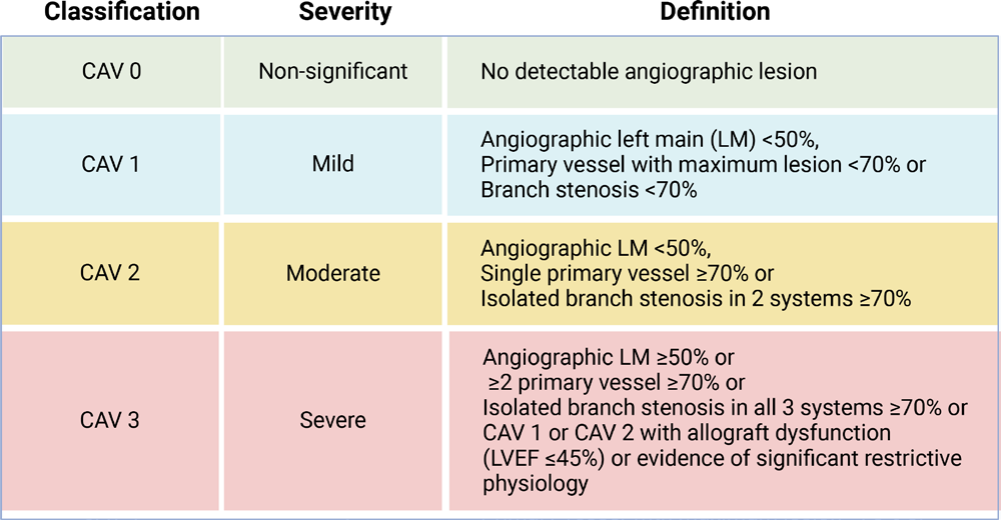
ISHLT recommended standardized CAV grading. CAV, cardiac allograft vasculopathy; ISHLT, International Society for Heart and Lung Transplantation; LVEF, left ventricular ejection fraction.

ICA can visualize the coronary arteries but can only measure the luminal diameter. ICA compares the luminal narrowing at sites with plaques to normal reference diameters to quantify the severity of the disease.^3,6,13^ CAV is often underestimated using ICA, since CAV often involves the entire length of coronary arteries and ICA may not detect a difference in luminal diameter if there is diffuse disease.^3,4,6,13,14^ However, ICA also allows for functional assessment using the quantitative flow ratio, which evaluates the impact of stenosis by analyzing blood flow and pressure. Grading of angiographic severity of CAV is based on anatomical diameter stenosis and standardized with ISHLT scores ranging from ISHLTCAV0 to ISHLT CAV3 as detailed in Figure 1.^2^ CAV is detected by ICA in 30%–50% of the cases 5 y after HTx and 20% of patients exhibit moderate to severe disease, categorized as ISHLT CAV2-3, within 12 y after HTx.^1,2,14,15^ It is important to acknowledge that in the transition from CAV0 to CAV1, a considerable amount of disease can already be present.

IVUS allows for the simultaneous evaluation of luminal diameter, the extent of intimal hyperplasia, and plaque morphology.^2,6,14^ Intimal thickening on IVUS can be seen as early as 6 wk after transplantation.^2^ Serial IVUS allows for the assessment of disease progression by quantifying the change in intimal hyperplasia.^2,14^ An increase in maximal intimal thickening of ≥0.5 mm during the first year post-HTx on IVUS has been associated with higher risks of mortality, non-fatal major adverse cardiac events, and the development of angiographic CAV within 5 y post-HTx.^1,16^ Unfortunately, like ICA, IVUS is invasive and only visualizes the major coronary arteries and thus cannot be used to screen the entire coronary vasculature. It also requires heparinization, which brings more risks to patients.^2,6,14^ Another limitation of IVUS is the need for (expensive) catheters, making it less easily accessible. In addition, due to the necessity for an additional catheter in the coronaries during IVUS, there is a higher risk of complications.^2^

## THE VALUE OF CT-BASED CAV SURVEILLANCE

### Assessment of CAV

**FIGURE 2.**
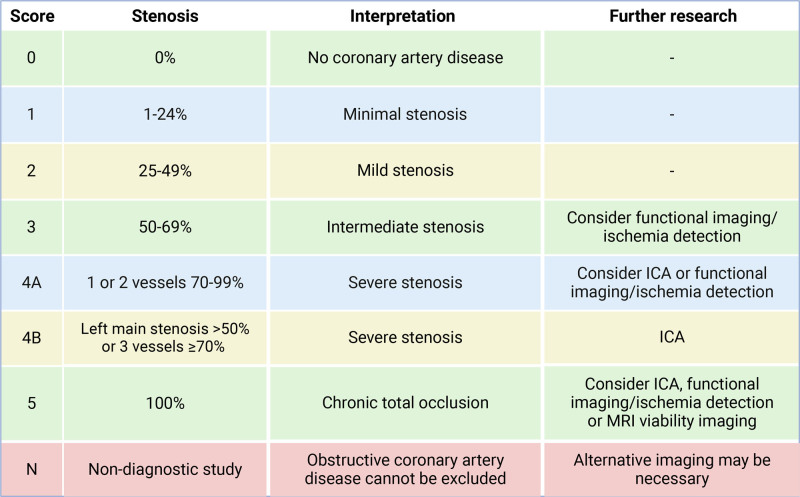
CAD-RADS 2.0 scoring for stenosis grading. CAD-RADS, Coronary Artery Disease Reporting and Data System; ICA, invasive coronary angiography.

**FIGURE 3.**
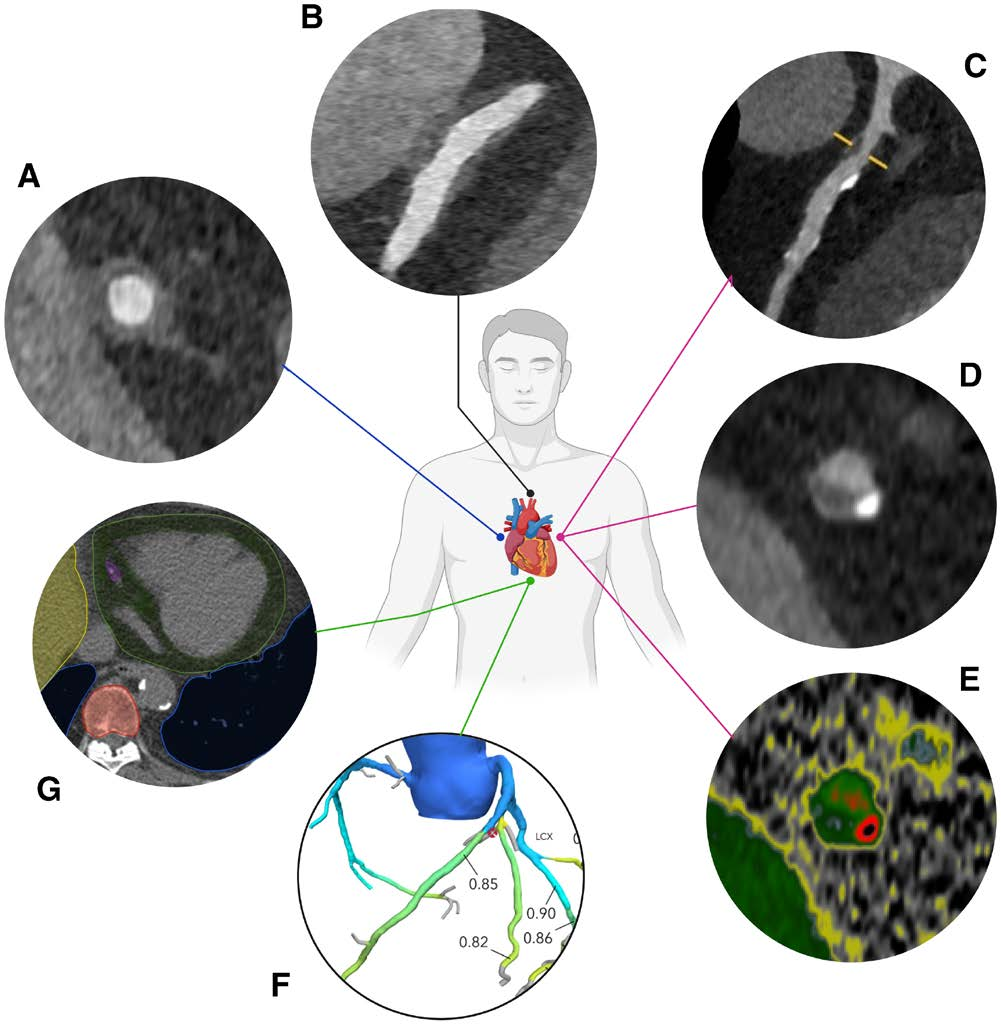
Assessments derived from coronary computed tomography (noncontrast and contrast-enhanced) angiography from an HTx patient. A, Diffuse thickening of the intima as seen in CAV. B, CAV plaque formation along the whole length of the coronary. C, Soft plaque and calcium are shown in the LAD coronary artery (yellow markers). D, Corresponding intersection of LAD with soft plaque and calcium. E, Plaque analysis of the corresponding intersection of the LAD: lumen (green), soft plaque (grey), coronary calcium (red and black). F, Fractional flow reserve CT from an HTx patient. G, Cardiometabolic and general health markers, as seen on a noncontrast CT scan: coronary artery calcium (purple), epicardial fat (green), left liver lobe (yellow), thoracic vertebra (red), and lung tissue (blue). CAV, cardiac allograft vasculopathy; CT, computed tomography; HTx, heart transplant; LAD, left anterior descending.

Coronary CT angiography (CCTA) is an attractive noninvasive technique for CAV surveillance with excellent sensitivity, specificity, and negative predictive value (NPV).^1,2,11,12,17-19^ Recent technological advancements in CCTA have significantly improved the diagnostic accuracy for evaluation of any CAV stenosis, as shown in Table 1.^11,18,20-32^ Using CCTA to detect luminal irregularities versus significant CAV (lumen stenosis ≥50%) shows sensitivities of 97% and 94%, specificities of 81% and 97%, and NPVs of 95% and 99%.^11,19^ Comparing CCTA versus ICA, which is considered the gold standard, for the detection of significant CAV (lumen stenosis ≥50%) shows a sensitivity of 94%, specificity of 92%, and NPV of 99%.^33^ The sensitivity and NPV of CCTA compared with ICA to detect CAV is significantly better.^11,19,33^ As previously noted, the use of the ISHLT CAV grading system is associated with a significant difference in disease severity between CAV0 and CAV1.^2^ The Coronary Artery Disease Reporting and Data System (CAD-RADS) 2.0 scoring system, a radiological methodology for evaluating stenosis in coronary segments, can be integrated with CCTA, as can be seen in Figure 2.^34^ With ISHLT CAV grading, a CAV0 to CAV1 classification is defined as an angiographic left main stenosis of <50%, or stenosis of a primary vessel with a maximum lesion of <70%, or branch stenosis of <70% (Figure 1).^2^ In contrast, CAD-RADS 2.0 assigns a score even for lesser degrees of stenosis, with 1%–24% and 25%–49% stenosis receiving distinct scores. This offers a more gradual grading, enabling a more precise analysis of disease progression, thus facilitating earlier and more targeted treatment.^34^ However, the CAD-RADS 2.0 has not been used in HTx patients. The clinical value of the scoring system in this patient category needs to be evaluated. ICA, functional imaging, or MRI viability imaging may all be considered (Figure 2). As previously noted, maximal intimal thickening detected by IVUS is a reliable predictor of mortality.^1,16^ CCTA could potentially be used as a noninvasive alternative for measuring wall thickening, especially when using ultra-high-resolution CT. However, the clinical value to predict mortality of this noninvasive alternative still needs to be processed. To thoroughly evaluate cardiac health using CCTA, it is essential to follow a standardized protocol that involves both contrast and non-contrast scans specifically for assessing coronary artery calcium (CAC) and non-calcified plaques. Additional opportunities of CCTA (depending on the scan protocol) include the ability to analyze wall motion, inflammation of pericoronary fat, quantitative plaque analysis, and stress perfusion, for which intravenous adenosine or adenosine derivatives, such as regadenoson, are necessary.^1,2,12,13,35^ Quantitative plaque analysis enhances the identification of early CAV and facilitates the monitoring of CAV progression over time, as can be seen in Figure 3.^33^ The incorporation of this analysis alongside visual interpretation increases the sensitivity of CAV detection with CCTA without reducing specificity.^30^ Patients with CAV exhibit a higher prevalence of non-calcified plaques compared with calcified plaques. Additionally, patients with a history of CAV have higher volumes of non-calcified plaques compared to other HTx patients, suggesting a potential risk factor for patients with more rapidly progressive CAV.^30^

**TABLE 1.** Overview of studies that assessed the diagnostic accuracy of CCTA for evaluation of the presence of any CAV post-HTx using a segment-based analysis

Study	Year	Study type	Reference standard	N	Time since HTx, mo	CT scanner (no. of slices)	Sensitivity, %, (95% CI)	Specificity, %, (95% CI)	PPV, %, (95% CI)	NPV, %, (95% CI)	Accuracy, %, (95% CI)
Romeo et al^20^	2005	Prospective	ICA	44	91	16	90 (69-97)	80 (60-91)	77 (56, 90)	91 (72, 98)	84 (71, 92)
Iyengar et al^21^	2006	Prospective	ICA	19	NR	64	100 (72-100)	67 (35-88)	77 (50-92)	100 (61-100)	84 (62- 96)
Sigurdsson et al^22^	2006	Prospective	IVUS	53	NR	16	96 (87-99)	88 (80-93)	80 (69-88)	98 (93-99)	91 (85-94)
Gregory et al^23^	2006	Prospective	IVUS	20	70	64	70 (57-80)	92 (83-97)	89 (76-95)	77 (67-85)	82 (74-88)
Usta et al^23, 24^	2009	Prospective	ICA	10	73	16	100 (51-100)	100 (61-100)	100 (51-100)	100 (61-100)	100 (72-100)
Schepis et al^25^	2009	Prospective	IVUS	30	34	64	85 (72-93)	84 (74-91)	76 (62-86)	91 (81-96)	85 (77-90)
Mittal et al^26^	2013	Prospective	ICA	130	144	64	98 (90-100)	80 (70-88)	78 (69-86)	98 (91-100)	88 (81-92)
Nunoda et al^30^	2010	Prospective	ICA	22	418	64	90	98	82	99	–
Miller et al^29^	2019	Retrospective	ICA	17	72	DSCT	70	100	–	–	–

Study	Year	Study type	Assessments on CCTA	N	Time since HTx, mo	CT scanner (no. of slices)	Main finding				
Nous et al^17^	2021	Prospective	Agatston calcium score, CAV grade	129	132	DSCT	CCTA can be successfully implemented for routine detection of CAV with good image quality and low radiation dose. CCTA allows CAV evaluation with the limited for additional invasive testing				
Budde et al^28^	2022	Prospective	FFRct analysis	73	132	DSCT	FFRct performed on CCTA scans of HTx patients demonstrated that 25% of patients had focal coronary stenosis with FFRct ≤0.8. Even without focal stenosis, FFRct values are often abnormal in HTx patients				
Sharma et al^27^	2024	Retrospective	FFRct analysis, CAV grade	106	108	256-slice, DSCT or photon-counting	FFRct is important for monitoring CAV in heart transplant patients. Over time, transplant patients showed a decrease in distal FFRct and an increase in coronary stenoses. Temporal changes in FFRct could be crucial for transplant follow-up, aiding in CAV detection				
Wall et al^31^	2024	Retrospective	PCAT density	94	31	64-slice or DSCT	HTx patients with CAV have higher PCAT density than those without. Increased PCAT density is associated with adverse clinical outcomes. These CCTA metrics could be useful for the diagnosis and monitoring of CAV severity				

The upper part of this table is derived from Wever-Pinzon et al.^11^

CAV, cardiac allograft vasculopathy; CCTA, coronary computed tomography angiography; CT, computed tomography; DSCT, dual-source computed tomography; FFRct, fractional flow reserve computed tomography; HTx, heart transplantation; ICA, invasive coronary angiography; IVUS, intravascular ultrasound; NPV, negative predictive value; NR, not reported; PCAT, pericoronary adipose tissue; PPV, positive predictive value.

### Fractional Flow Reserve

Not only the anatomical features of the coronary vasculature can be seen on CCTA but also noninvasive functional assessment is possible, even in patients with a relatively high heart rate. Fractional flow reserve (FFR) is an additional analytical modality that can be performed on previously acquired CT images.^29^ In a more recent study of our group, HTx patients underwent FFRct analysis of CCTA for routine annual CAV screening. With these assessments, FFRct successfully identifies HTx patients with hemodynamically significant coronary stenosis, as can be seen in Figure 3.^29^

## ASSESSMENT OF CARDIOMETABOLIC HEALTH MARKERS

**FIGURE 4.**
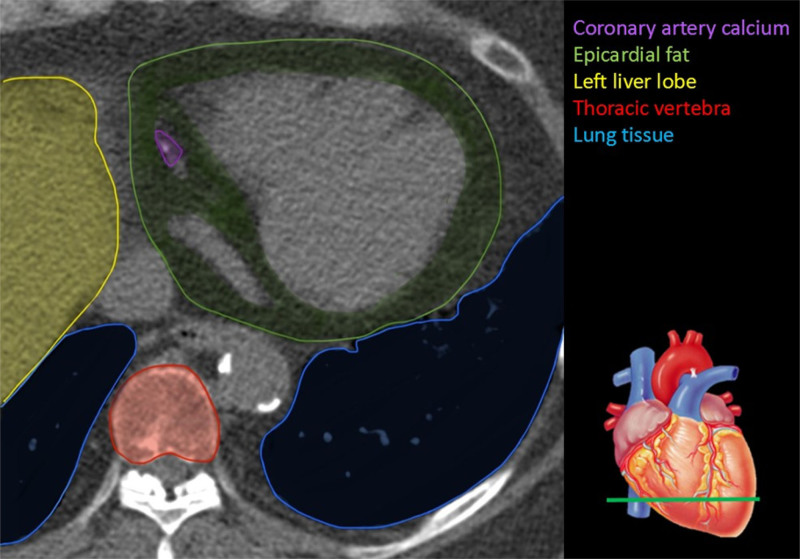
Imaging markers on a noncontrast coronary calcium scan with, in color, different tissues from which the potential imaging markers may be obtained.

**FIGURE 5.**
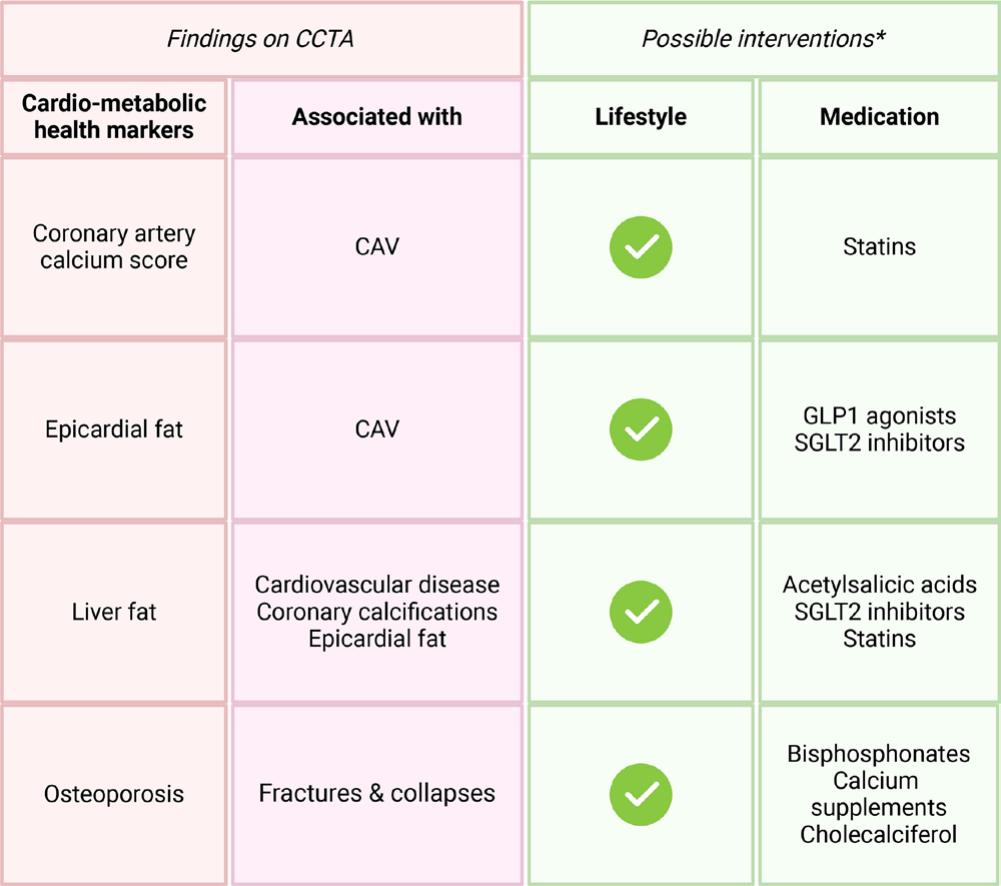
Clinical relevance of cardiometabolic health markers that can be found on CCTA. *There are multiple potential interventions available. These interventions have been proven effective in the general population beyond the context of transplantation. CAV, cardiac allograft vasculopathy; CCTA, coronary computed tomography angiography; GLP-1, glucagon-like peptide-1; SGLT2, sodium-glucose cotransporter 2.

Noteworthy, the yield of CCTA is expanded by its ability to derive a comprehensive set of emerging cardiometabolic markers in the field of HTx, such as CAC score, epicardial fat volume (EFV), and liver fat, as can be seen in Figure 4. To acquire the aforementioned measurements, a non-contrast scan must be incorporated into the CCTA scan protocol. The clinical relevance of these cardiometabolic health markers is shown in Figure 5.

### CAC Score

The CAC score, also referred to as the Agatston score, stratifies the general population and stable angina patients into low, intermediate, and high-risk groups concerning the future development of coronary artery disease.^36^ Furthermore, the spatial arrangement of calcification can be easily assessed, and the total CAC score offers prognostic insights concerning the likelihood of major coronary events. The CAC score is easy to measure on the noncontrast acquisition as all pixels in the coronary arteries with a Hounsfield Unit (HU) of >130 are included. In HTx patients, the presence of any CAC is significantly associated with a negative outcome, with more nonfatal major adverse cardiac events and more deaths or graft losses compared with HTx patients without coronary calcifications.^37^ On the other hand, the absence of CAC predicts a low prevalence of CAV in HTx patients, with an NPV of 97% for ISHLT CAV 2-3.^37^ Whether or not CAC is a therapeutic target in CAV in HTx patients has not been prospectively evaluated, but with the increasing role for CCTA in CAV, the CAC will become available for physicians and for example, personalized, CAC-based lipid-lowering regimens may be evaluated for their long-term benefits.

### Epicardial Fat Volume

Epicardial fat is the layer of metabolically active adipose tissue encircling the myocardium and coronary arteries. In general population cohorts, associations of larger EFV with increased risk of coronary atherosclerosis became evident.^38-40^ Post-HTx patients appear to be accumulating more EFV compared with the general population, which indeed may translate to higher CAV grades in HTx patients.^38^ Future research should be conducted to ascertain whether EFV underlies the accelerated onset of CAV in HTx patients. Recent advancements in image processing techniques have aided the appraisal of epicardial fat from non-contrast CT scans.^35^ Sodium-glucose cotransporter 2 inhibitors and glucagon-like peptide-1 agonists have demonstrated the capacity to decrease EFV in the general population.^38,41,42^ Proprotein convertase subtilisin/kexin type 9 inhibitors have been suggested as a potential pharmacological approach to reduce epicardial fat.^38,43,44^ Further research is needed to validate that EFV can be reduced in HTx patients and to assess whether this could decelerate CAV progression.^38,45^

### Liver Fat

The upper part of the liver is often in the scan range of a CAC scan. Measuring the mean HU values at various locations in the liver is a reliable marker of the overall fat content of the liver, with lower HU values reflecting larger amounts of liver fat.^35,46^ The amount of liver fat holds significant importance, as it is associated with cardiovascular disease.^35,46^ Increased liver fat correlates with higher levels of epicardial fat and atherosclerotic calcifications, independent of typical cardiovascular risk factors.^35^ Additionally, liver fat is related to adverse cardiac remodeling.^35,46,47^ Measuring the liver fat is a way to monitor the cardio-metabolic health of patients and intervene when necessary. As with CAC, we have no prospective data to support specific treatments based on liver fat content, so measuring liver fat currently has no clinical implications.

## ASSESSMENT OF OTHER GENERAL HEALTH MARKERS

The combination of all measurements mentioned previously comprises a comprehensive cardio-metabolic evaluation that is derived from a CCTA scan. The obtained information can be further expanded by deriving general health markers, such as vertebral bone and lung density.^1,2,12,13,35,38^

### Vertebral Bone Density

Post-transplant patients use steroids for prolonged periods, which is a risk factor for developing osteoporosis. Identifying osteoporosis is very important for the prevention, prediction, and management of the condition.^48^ Bone mineral density (BMD) is a key modifiable risk factor for osteoporotic fractures and the existence of vertebral osteoporotic fractures.^35,38,48^ Lumbar quantitative CT uses a 3-dimensional approach offering detailed insights into volumetric BMD and independent characterization of bone geometry, enhancing fracture risk evaluation.^49^ Lumbar quantitative CT exhibits high sensitivity for diagnosing and monitoring osteoporosis, but it is rarely used to assess BMD due to higher radiation and costs.^35,48,49^ However, thoracic BMD can also be accurately and precisely evaluated during routine CCTA scans, as these scans typically cover multiple thoracic vertebrae, allowing for the assessment of thoracic BMD^35,38,48^ (Figure 4). Thoracic BMD is assessed on CCTA by measuring HU at 3 consecutive thoracic vertebrae. The assessment is initialized from the slice level containing the left main coronary artery and proceeds caudally. Cortical bone, venous vessels, and fractures are excluded using a manual tracing protocol. HU values for the vertebrae can be automatically displayed. The calculation of mean BMD values for the 3 thoracic vertebrae is subsequently performed.^48^

### Lung Density

Emphysema can be assessed on a CT scan. Assessing lung density as a direct indicator of emphysema can present challenges, particularly due to the constrained field of view in most scans, designed primarily to visualize the heart. Generally, the overall lung density assessment can be performed within the lower lung lobes and the regions adjacent to the hila. An alternative approach involves considering the reconstruction of the CAC scan using a wider field of view, visualizing the entirety of the original lung tissue within the scan. Although the upper lung regions will still be missing, this method accurately evaluates the remaining part of the lungs concerning emphysema presence. This approach can also facilitate the identification of other lung-related observations, such as possibly malignant pulmonary nodules.^35,50,51^ Incidental findings in the lung are possible to detect on a CAC scan.^35^ Extending the scan range craniocaudally in the non-contrast scan should be considered. This slight adjustment allows for a comprehensive assessment of the entire lung for potential malignancies.

## PHOTON-COUNTING CT

Photon-counting CT (PCCT) is an innovative imaging technique that provides improved spatial resolution to regular energy-integrating detector CT scanners, as well as spectral imaging capabilities and noise reduction.^52^ This allows for improved visualization of small arteries, stents, calcifications, and noncalcified plaques compared with conventional CCTA.^20,52-55^ In conventional CCTA, metal stents often appear thicker because of blooming artifacts, which hinder the assessment of the in-stent lumen. PCCT minimizes blooming artifacts, resulting in more precise measurements of the in-stent diameter.^20,52-54^ The same holds for blooming artifacts caused by calcifications.^20,52-54^ Furthermore, PCCT can detect smaller and less dense calcifications and allows visibility of dense calcifications at a lower radiation dose than conventional CCTA. The increasing usage of PCCT could lead to changes in acquisition protocols, including reduced radiation doses for precise imaging.^52,56,57^ Additionally, PCCT offers sharper calcification borders, resulting in enhanced calcification detection.^20,52-57^ These assessments can enable the monitoring of interventions with PCCT to determine their efficacy.

## IMPLEMENTATION OF CCTA-BASED CAV SURVEILLANCE

A potential obstacle to the implementation of CCTA as standard practice for CAV surveillance is the associated radiation exposure of 2.1–4.1 mSv.^58^ However, this is less radiation exposure compared with ICA, which ranges from 4.1 to 5.6 mSv.^58^ CCTA can evaluate coronary vessels down to approximately 1 mm in diameter. However, it still faces limitations in assessing smaller vessels and microvasculature.^2,3,13,14,18^ Additionally, there is a risk of contrast-induced nephropathy when using CCTA. The volume of iodinated contrast used in CCTA likely varies between hospitals, but the typical volume used is approximately 60 mL. There are currently no alternatives to iodinated contrast agents for CCTA.^2,3,13,14,18,58^ Another obstacle is possible motion artifacts due to higher resting heart rates in HTx patients.^2,3,13,14^ Recent studies have demonstrated that a higher resting heart rate has little to no effect on image quality with modern dual-source CT scanners.^53^ However, intravenous beta-blockers are given if deemed necessary (heart rate ≥70/min) up to the dosage authorized by the transplant cardiologist who requested the CCTA.^18^ To ensure diagnostic image quality, patients receive sublingual nitroglycerin before undergoing CCTA. Furthermore, modern CT scanners can reduce the radiation dose for precise imaging CCTA, which compares favorably with ICA in detecting CAV and may be a preferable screening technique because of the primary advantages, such as the direct visualization of the coronary arteries with less patient discomfort, fewer risks, and costs.^1,2,17^

With the implementation of CCTA-guided CAV surveillance, it is necessary to have effective treatment options if CAV is detected. The impact of stenting in managing CAV is possibly limited because the ISHLT CAV grading system only prompts coronary interventions at higher CAV grades.^1^ Using the CAD-RADS 2.0 scoring system allows for timely interventions, such as adjusting or introducing pharmacological treatments. However, the question remains whether this is effective in delaying the progression to more severe disease stages.^34^ Standard cardiovascular risk management typically consists of increasing statin dosage, stricter regulation of diabetes mellitus, and managing hypertension as well as renal insufficiency. This approach can be further optimized by incorporating mammalian target of rapamycin inhibitors, such as everolimus, in accordance with HTx management protocols.^2^ The impact of adjusting or introducing pharmacological treatments on survival should be investigated.

Another area of uncertainty concerns the implementation of CCTA-based CAV surveillance. Currently, only expert opinions are available. At our center, we initiate baseline CCTA before discharge post-HTx and conduct annual follow-up from 5 y post-HTx. Depending on the results, we may refer patients for FFR or additional ICA. Meanwhile, per clinical protocol, we conduct ICA at 1 and 4 y post-HTx.

## OTHER POSSIBILITIES FOR CAV SURVEILLANCE

**FIGURE 6.**
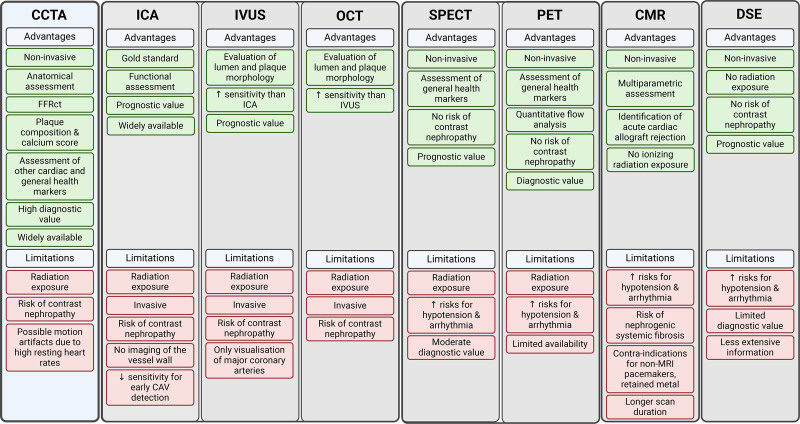
Comparison of CCTA to other imaging modalities. CCTA, coronary computed tomography angiography; CMR, cardiovascular magnetic resonance; DSE, dobutamine stress echocardiography; ICA, invasive coronary angiography; IVUS, intravascular ultrasound; OCT, optimal coherence tomography; PET, positron emission tomography; SPECT, single-photon emission computed tomography.

Various other imaging tools are available to screen HTx patients for CAV. Advanced imaging modalities such as optimal coherence tomography (OCT), single-photon emission CT (SPECT), Positron emission tomography (PET), cardiovascular magnetic resonance (CMR), and dobutamine stress echocardiography (DSE) have been examined for their potential use. The comparison of CCTA to other imaging modalities is shown in Figure 6.

These imaging modalities offer diagnostic or prognostic value, evaluation of lumen and plaque morphology, and comprehensive assessments of myocardial perfusion.^1-3^ PET can detect microvascular disease with flow quantification, and CMR can identify acute CAV rejection.^59,60^ Non-invasive modalities, for example, CMR and DSE, are beneficial for patients with contraindications to invasive procedures or contrast administration.^1,17^

However, these techniques are less likely to be incorporated as standard practice for CAV surveillance due to various limitations. For instance, high costs, limited accessibility, and the need for specialized equipment and trained personnel are significant barriers.^1-3,17,59^ The need for additional contrast administration with OCT can hinder accessibility.^1-3^ SPECT, PET, CMR, and DSE require the administration of intravenous dobutamine, adenosine, or adenosine derivatives, which can lead to complications such as arrhythmia and hypertension.^1-3,17,59-61^ Additionally, certain techniques have limitations in diagnostic accuracy. The limited tissue penetration of OCT restricts the assessment of deep plaques, SPECT has a limited ability to detect balanced ischemia, and DSE provides less extensive information than invasive techniques.^1-3,17^

Although these imaging modalities present valuable tools for CAV surveillance, their integration into standard practice is hindered by economic, logistical, and technical challenges.

## DISCUSSION

CCTA compares favorably to other imaging modalities as a non-invasive technique for CAV surveillance. Moreover, CCTA can be an excellent gatekeeper to assess whether a patient needs ICA due to the high NPV. CCTA offers a comprehensive assessment of the coronary arteries, which is crucial for CAV surveillance. The incorporation of FFRct provides insights into hemodynamically significant coronary stenosis, effectively identifying patients requiring intervention. The multifaceted approach of CCTA extends by deriving cardio-metabolic markers, such as CAC score, epicardial fat, and liver fat. CCTA goes beyond cardiovascular assessment by evaluating general health markers like vertebral bone density, lung density, and pulmonary nodules. The ability of CCTA to assess cardiovascular, cardio-metabolic, and general health markers in a single scan protocol, while offering diagnostic value, positions CCTA as an attractive choice for effective CAV monitoring and patient care for HTx patients.

Potential obstacles to using CCTA as standard surveillance for CAV include radiation exposure, contrast-induced nephropathy, and motion artifacts. However, the primary advantages, including direct visualization of the coronary arteries with less patient discomfort, lower risks, and lower costs compared with conventional ICA, outweigh the concerns.

Alternative techniques for CAV surveillance, such as OCT, DSE, SPECT, CMR, and PET, have limitations in terms of accuracy, diagnostic value, cost, or feasibility. The accessibility of OCT is restricted because of high costs and limited tissue penetration. The limited diagnostic value of DSE and the safety concerns in HTx patients prevent broader use. The low to moderate accuracy of SPECT and the inability to detect balanced ischemia limits diagnostic utility. The high costs and limited availability of PET and CMR hinder clinical implementation.

The utilization of CCTA demonstrates promising implications for future medical applications. With the advent of PCCT, there is a potential for a reduction in radiation dose, accompanied by an enhanced capability for comprehensive assessment. Due to the high spatial resolution and spectral imaging capabilities available with PCCT, the heightened precision in detecting smaller calcifications and in-stent stenosis provides a promising outlook on the evaluation of smaller vessels and patients with previous PCI. More research is needed to further describe the impact that PCCT may have on optimal CAV detection. The current capacity already positions CCTA as a method capable of detecting early stages of CAV, preceding other modalities, thereby allowing early intervention and delaying the progression of CAV. Timely administration of medication, facilitated by the early detection afforded by CCTA, holds the potential to mitigate the progression of CAV. Clinical cardiologists, cardiovascular radiologists, and medical specialists in this field should be aware of the advantages and clinical relevance of CCTA.
